# Extensive dental caries and periodontal disease in a child with GATA2 deficiency

**DOI:** 10.4317/jced.60345

**Published:** 2023-09-01

**Authors:** Filippo Consonni, Eleonora Gambineri, Marinella Veltroni, Michele Callea

**Affiliations:** 1 MD. Department of Health Sciences, University of Florence, Florence, Italy; 2 MD. Centre of Excellence, Division of Paediatric Oncology/Haematology, Meyer Children’s Hospital IRCCS, Florence, Italy; 3 MD. Department of Neurosciences, Psychology, Drug Research and Child Health (NEUROFARBA), University of Florence, Florence, Italy; 4 DDS, MoH, MSc. Meyer Children’s Hospital IRCCS, Paediatric Dentistry and Special Dental Care Unit

## Abstract

**Background:**

GATA2 deficiency is an inborn error of immunity (IEI) characterized by infectious susceptibility and increased risk of myelodysplasia leading to acute myeloid leukaemia (AML). Oral anomalies already described in this disorder include recurrent viral and fungal infections and oral ulcers.

**Material and Methods:**

We report a 9-year-old girl presenting with AML with myelodysplasia-related changes, monosomy 7 karyotype on marrow aspirate, numerous flat warts on her hands and multiple dental caries at oral cavity inspection. Dental evaluation and genetic testing (Sanger sequencing) for GATA2 were carried out considering the peculiar clinical presentation.

**Results:**

Dental evaluation showed extensive caries and periodontal disease, while genetic studies revealed a known c.1009 C>T (p.Arg337X) mutation in GATA2. After multidisciplinary discussion, affected teeth were extracted before chemotherapy, in general anaesthesia, together with scaling and root planning of the alveolar sockets. Subsequently, the patient underwent hematopoietic stem cell transplantation (HSCT) from her HLA-matched GATA2 wild-type sibling, who did not bear any dental anomalies. No dento-alveolar infections were encountered during post-chemotherapy aplasia.

**Conclusions:**

This case first describes the association between GATA2 deficiency and extensive dental caries with periodontal disease, highlighting the importance of an early dental evaluation and intervention in children with leukaemia.

** Key words:**GATA2 deficiency, Inborn errors of immunity, teeth, dental decay, multidisciplinary approach.

## Introduction

GATA2 deficiency (MIM#614172) determines a syndrome characterized by a wide range of clinical manifestations including myelodysplasia leading to acute myeloid leukaemia (AML), monocytopenia and mycobacterial infections (MonoMAC) syndrome, diffuse warts and lymphedema (1,2). Infectious manifestations and the deficiency of NK, B and dendritic cells conFigure this disorder as an Inborn Error of Immunity (IEI) (3,4). GATA2 deficiency is characterized by variable expressivity and phenotypic diversity. Among this pleiotropic clinical heterogeneity, oral manifestations due to fungal or viral infections are not rare. Similarly, dental anomalies frequently occur in other IEI (5-10). However, dental caries have never been specifically described in GATA2 deficiency (1,3,5,6). Herein, we report a child with GATA2 deficiency, who presented to our Dentistry unit for an evaluation in the context of AML.

## Case Report

**Figure 1:**
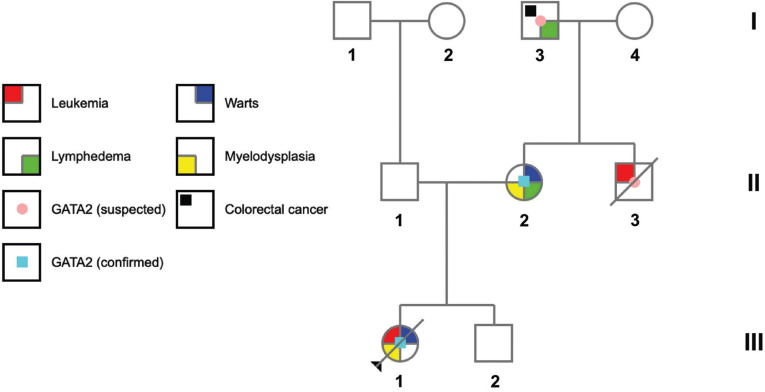
Family pedigree of the reported patient (III.1, arrowhead). A maternal uncle (II.3) had died of leukemia in adolescence, the mother (II.2) suffered from leg lymphedema and recurrent skin warts, while the maternal grandfather (I.3) also displayed lymphedema, together with colorectal adenocarcinoma. GATA2 deficiency was genetically confirmed in the patient and her mother. Family members with a strong suspicion of GATA2 deficiency considering the clinical history are reported.

A 9-year-old girl was referred to our Haematology-Oncology Unit from a foreign country for severe anaemia (Hb 3,9 g/dl, MCV 101 fl) with low monocytes and reticulocytes. Family history revealed peculiar clinical features on the mother’s side (Fig. 1). At clinical evaluation, the girl displayed pale skin, numerous flat warts on her hands, while oral cavity inspection revealed multiple dental caries.

Bone marrow biopsy and aspirate were performed, showing 30-35% marrow infiltration by immature myeloblasts, defining AML with myelodysplasia-related changes. Cytogenetic studies showed the presence of monosomy 7 karyotype on marrow aspirate. In light of the family history and the clinical and laboratory features, Sanger sequencing of *GATA2* was performed, showing a heterozygous, maternally-inherited, c.1009 C>T (p.Arg337X) mutation, already known as pathogenic (2).

**Figure 2:**
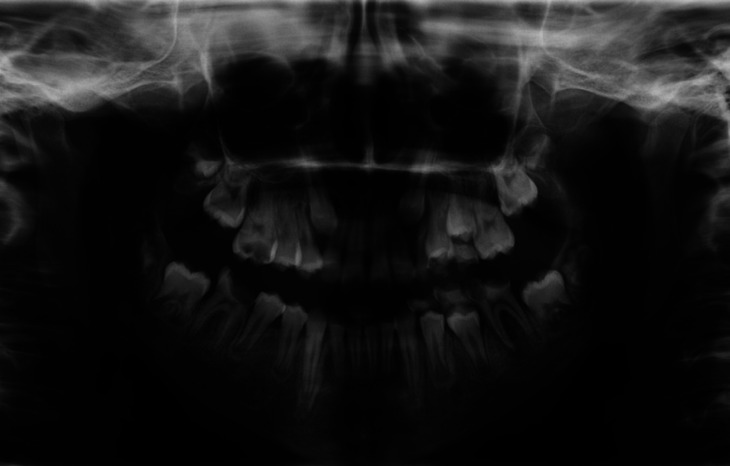
Orthopantomography showing mixed dentition and multiple dental caries in both primary and permanent dentition; periodontal diseases is also visible, along with crowding in the upper jaw leading to malocclusion.

Moreover, a dental evaluation and orthopantomography were carried out, showing deep decay on several primary and permanent teeth, respectively 6.3, 6.4, 6.5, 7.4, 7.5, 1.6, 3.6 and 4.6. Vertical and horizontal bone resorption was visible on the orthopantomogram (Fig. 2) as well as in the intraoral examination. The patient’s collaboration was almost absent, hence – after a multidisciplinary discussion with the Oncology-Haematology Unit – the decision of performing a multiple dental extraction under general anaesthesia was made, in order to reduce the risk of infections in a potential candidate to haematopoietic stem cell transplantation (HSCT) and in light of the high chance of oro-dental complications during AML treatment (11).

We performed a surgical extraction of all the above-mentioned teeth, placing resorbable suture and platelet foam gel, as well as tranexamic acid in 4.8% concentration used both topically and intravenously. In fact, due to the severely compromised periodontal status (despite the young age of the patient) and due to the deep scaling and root planning carried out during the operation, the bleeding had been quite considerable.

The patient underwent 2 cycles of Idarubicin-Cytarabine-Etoposide chemotherapy according to local AML treatment protocol. Marrow evaluations after chemotherapy showed poor response to treatment, therefore – despite the persistence of myeloblasts at marrow re-evaluation –, the family accepted to undergo HSCT from the patient’s HLA-matched, GATA2 wild-type sibling. Of note, no dento-alveolar infections were encountered during chemotherapy and her healthy brother did not bear any teeth abnormalities.

HSCT was performed upon myeloablative conditioning (Thiotepa-Treosulfan-Fludarabine) using bone marrow as stem cell source. No severe infections took place during post-induction aplasia, and engraftment was successfully attained with full donor chimerism. Post-transplant follow-up showed grade I upper gastro-intestinal Graft-versus-Host Disease, treated with topical steroids. Unfortunately, AML recurrence occurred and the patient eventually deceased 4 months after HSCT.

## Discussion

To our knowledge, this is the first case of extensive caries and periodontal disease in a patient with GATA2 deficiency. Therefore, this report expands the spectrum of the oral manifestations of this disorder, which already include oral ulcers and recurrent fungal, HSV and HPV infections, that may eventually lead to squamous cell carcinoma (1,5,12,13).

The dental involvement of our patient was diagnosed at the onset of AML, which is notably associated with immune suppression and oral manifestations (11). However, given the slow timing of caries development (14), the dental phenotype of our case is not attribuTable to the ongoing leukaemia, which on the other hand has an extremely rapid growth rate (15). Educational factors (e.g., a lack of dental hygiene) and/or a familiar predisposition may undoubtedly have contributed to the pathogenesis of the oral disease, even though the absence of caries in her GATA2 wild-type brother implies that the underlying immunodeficiency played a leading role.

Despite the severity of the dental Tableau, an early surgical approach with extraction of the affected teeth with scaling and root planning of the alveolar sockets allowed to reduce the risk of life-threatening oral infections, especially throughout the prolonged phase of post-induction aplasia during HSCT. This suggests that pre-transplant work-up should regularly include a dental evaluation and eventually a preventive surgical intervention, especially in children more prone to develop oral disease, such as those affected by IEI (5,6,8).

In conclusion, we report the first case of GATA2 deficiency associated with dental caries and periodontal disease, expanding the clinical spectrum of this disorder.
